# Liver dECM–Gelatin Composite Bioink for Precise 3D Printing of Highly Functional Liver Tissues

**DOI:** 10.3390/jfb14080417

**Published:** 2023-08-09

**Authors:** Min Kyeong Kim, Wonwoo Jeong, Hyun-Wook Kang

**Affiliations:** Department of Biomedical Engineering, Ulsan National Institute of Science and Technology (UNIST), UNIST 50, UNIST-gil, Ulsan 44919, Republic of Korea; minkim93@kbsi.re.kr (M.K.K.); wjeong@wakehealth.edu (W.J.)

**Keywords:** bioink, 3D bioprinting, decellularized extracellular matrix, liver tissue engineering

## Abstract

In recent studies, liver decellularized extracellular matrix (dECM)-based bioinks have gained significant attention for their excellent compatibility with hepatocytes. However, their low printability limits the fabrication of highly functional liver tissue. In this study, a new liver dECM–gelatin composite bioink (dECM gBioink) was developed to overcome this limitation. The dECM gBioink was prepared by incorporating a viscous gelatin mixture into the liver dECM material. The novel dECM gBioink showed 2.44 and 10.71 times higher bioprinting resolution and compressive modulus, respectively, than a traditional dECM bioink. In addition, the new bioink enabled stable stacking with 20 or more layers, whereas a structure printed with the traditional dECM bioink collapsed. Moreover, the proposed dECM gBioink exhibited excellent hepatocyte and endothelial cell compatibility. At last, the liver lobule mimetic structure was successfully fabricated with a precisely patterned endothelial cell cord-like pattern and primary hepatocytes using the dECM gBioink. The fabricated lobule structure exhibited excellent hepatic functionalities and dose-dependent responses to hepatotoxic drugs. These results demonstrated that the gelatin mixture can significantly improve the printability and mechanical properties of the liver dECM materials while maintaining good cytocompatibility. This novel liver dECM gBioink with enhanced 3D printability and resolution can be used as an advanced tool for engineering highly functional liver tissues.

## 1. Introduction

The liver is an essential organ that performs important functions such as protein synthesis, blood detoxification, xenobiotic metabolism, and immune activity. It can be damaged by various factors such as stress, alcohol consumption, and viruses [1]. Currently, transplantation of healthy liver tissue is the only treatment available for chronic liver diseases, and only a few patients have successfully received treatment, owing to challenges such as donor shortage and immune compatibility. To address these problems, various hydrogel-based technologies have been developed for the fabrication of highly functionalized tissue [2,3]. Among these technologies, 3D bioprinting enables the production of precise patterns using various types of biomaterials and cells [4]. Therefore, bioprinting is one of the useful technologies for producing highly functional liver tissue. Bioink is an essential element in the bioprinting process because it directly affects both the patterning performance and the functionality of a bioprinted tissue construct. Bioinks for liver tissue engineering are typically prepared using commercially available biomaterials such as gelatin, collagen, and alginate [5,6,7,8]. User-designed hepatic structures have been fabricated using these biomaterials. However, these conventional bioinks are limited in providing a good biological environment for liver tissue fabrication because biochemical compositions similar to those of the in vivo liver tissue extracellular matrix (ECM) cannot be achieved using existing biomaterials. Accordingly, liver decellularized ECM (dECM)-based bioinks are receiving increased attention in liver tissue engineering studies [9].

The decellularization process removes cells and allows selective extraction of ECM material from native tissues [10]. dECM materials are mainly composed of collagen fibrils and tissue-specific chemical compositions that can provide a suitable biological environment for artificial tissue fabrication [11,12,13]. A technology that enables the development of bioinks with dECM materials for 3D bioprinting using pepsin digestion has been introduced [14,15,16,17]. It has enabled the development of dECM bioinks for 3D bioprinting technology using various types of tissues, including cardiac [14], skeletal muscle [15], cartilage [16], and pancreatic tissues [17]. Porcine liver-based dECM bioinks have also been introduced, and they have shown good performance in engineered liver tissue studies [18,19,20]. However, they have several limitations. During the pepsin digestion process, a denaturing phenomenon of liver dECM materials occurs, which significantly weakens the mechanical properties of liver dECM bioinks [21,22]. Therefore, conventional liver dECM bioinks have low 2D/3D printability, making it challenging to print a biomimetic cellular construct using them [23,24]. Furthermore, cell–cell and cell–ECM interactions have the greatest influence on the functionality of bioprinted liver tissues, and precise patterning technology using multiple types of cells is essential to significantly functionalize the interactions [25,26,27,28]. Accordingly, conventional dECM bioinks with low mechanical properties and printability have limitations in producing highly functional liver tissues.

In this study, we developed a novel liver dECM-based bioink, called dECM–gelatin composite bioink (dECM gBioink), with significantly improved 2D/3D printability. The dECM gBioink was prepared by incorporating a gelatin mixture and dECM material derived from porcine liver tissue. To investigate the properties of the dECM gBioink, mechanical properties were analyzed. Moreover, 2D/3D printability tests were conducted to confirm the printing resolution, 2D patterning performance, and multilayer stacking performance of the dECM gBioink. A cytocompatibility test using primary mouse hepatocytes (PMHs) and human umbilical vein endothelial cells (HUVECs) was performed to demonstrate the feasibility of the proposed bioink in liver tissue engineering. Finally, a liver lobule-like structure with proper hepatic functionality was fabricated and subjected to liver toxicology testing. Using these experiments, we demonstrated that the proposed dECM gBioink has an enhanced potential for use in the precise bioprinting of highly functional liver tissues.

## 2. Method

### 2.1. Preparation and Analysis of Liver dECM

Porcine liver tissues were purchased from a slaughterhouse and sliced to 1.5 mm thickness. For decellularization, the sliced tissues were treated with 1% *v*/*v* Triton X-100 (Samchun Chemical Co., Ltd., Seoul, Republic of Korea) and 0.1% *v*/*v* ammonia solution (Samchun Chemical) for 24 h and then washed with distilled water for 48 h. Decellularization was performed by gently shaking the tissues at 4 °C.

Histological analyses and biochemical assays were conducted to evaluate the decellularized tissues. For the histological analyses, native and decellularized liver tissues were fixed with 4% *v*/*v* paraformaldehyde (PFA, Samchun Chemical) embedded in paraffin, and then sectioned at 5 μm thickness using a microtome (Leica, Bensheim, Germany). The sectioned tissue slides were stained with hematoxylin and eosin (H&E, Abcam, Cambridge, UK), Hoechst 33342 (H33342, Sigma, St. Louis, MO, USA), and Masson’s trichrome (Polysciences Inc., Warrington, PA, USA) after deparaffinization. The DNA content in the liver dECM was quantified using genomic DNA extraction [23]. In detail, the native and decellularized liver tissues were briefly lysed with a Tris-EDTA buffer (Bioneer, Seoul, Republic of Korea) comprising 1% *v*/*v* sodium dodecyl sulfate (SDS, Bioneer) and 1.0 mg/mL proteinase K (Bioneer). Genomic DNA was precipitated by treating the tissues with 5 M NaCl (Samchun Chemical) and dissolving them in distilled water. Thereafter, the DNA concentrations in the tissues were measured using a Nanodrop Spectrophotometer (Thermo Fisher Scientific, Waltham, MA, USA). A Blyscan sGAG assay kit (Biocolor Life Sciences, Carrickfergus, UK) and a QuickZime Total Collagen assay kit (QuickZime Biosciences, Leiden, The Netherlands) were used according to the manufacturer’s instructions to determine the glycosaminoglycan (GAG) and collagen contents of the liver tissues, respectively.

### 2.2. Preparation of the Bioinks

The traditional dECM bioink was prepared using pepsin digestion. Liver dECM (1 g) was digested for 48 h at 18 °C using 0.1 N HCl solution (Sigma) supplemented with pepsin (100 mg, porcine pepsin, Sigma). The pH of the digested solution was then adjusted using 5 N NaOH solution (Sigma), and then 10% *v*/*v* of 10X phosphate-buffered saline (PBS, Sigma) was added. The traditional liver dECM bioink was prepared at concentrations of 2% and 4% *w*/*v*. The 4% *w*/*v* traditional dECM bioink was used to prepare the dECM gBioink. A collagen (Atellocollagen type I, Dalim Tissen, Seoul, Republic of Korea) hydrogel was prepared using the same digestion process. The traditional dECM bioink and collagen hydrogel were preserved at 4 °C. To prepare the gelatin mixture, hyaluronic acid (HA; 6 mg/mL, Sigma) was dissolved in a minimum essential medium (MEM, Corning, New York, NY, USA) overnight at 37 °C using a rotator. Thereafter, gelatin (75 mg/mL, Sigma) and fibrinogen (6 mg/mL, Sigma) were dissolved in the HA solution at 37 °C for 1 h. The prepared gelatin mixture was sterilized using a filter (0.45 μm, Sigma) and stored at −80 °C. This prepared gelatin mixture was mixed with the same volume of MEM to prepare gelatin-based bioink having HA (3 mg/mL), gelatin (37.5 mg/mL), and fibrinogen (3 mg/mL). Lastly, the liver dECM gBioink comprising HA (3 mg/mL), gelatin (37.5 mg/mL), fibrinogen (3 mg/mL), and dECM (2% *w*/*v*) was prepared by mixing the 4% *w*/*v* traditional dECM bioink and gelatin mixture at the same volume ratio.

### 2.3. Measurements of Rheological and Mechanical Properties of the Bioinks

The rheological properties of the prepared dECM bioinks were investigated. A shear sweep analysis (shear rate of 0.1–100 S^−1^ and 18 °C) was conducted to measure the viscosities of bioinks using the HAAKE MARS III Rheometer (Thermo Fisher Scientific). The gelatin-based bioink and dECM gBioink were thermally crosslinked with incubation at 4 °C for 10 min and then applied to the rheometer. The traditional dECM bioink was applied in the form of a pre-gel solution. A temperature sweep analysis (+4 °C/min, 4–37 °C, 2% strain, and 1 Hz frequency) was performed to identify the temperature-dependent crosslinking properties of the dECM bioinks using a Kinexus pro+ rheometer (Malvern Panalytical, Malvern, UK). A dynamic frequency sweep test (0.1–10 rad·s^−1^, 2% stain, and 37 °C) was conducted to measure the storage and loss modulus of the dECM bioinks using a Kinexus pro+ rheometer (Malvern Panalytical). Fully crosslinked dECM bioinks were used for dynamic testing.

The compressive modulus of the bioinks was measured using a universal testing machine (Instron Model 3342; Illinois Tool Works Inc., Glenview, IL, USA). The bioinks were fully crosslinked and punched into cylindrical shapes (1 mm height, 5 mm diameter). The punched specimens were loaded into a testing machine and compressed at a rate of 1 mm/min. After plotting the strain–stress curves, their compressive modulus was measured by calculating the slope of the stress–strain curve at 10% strain.

### 2.4. Scanning Electron Microscopy

Scanning electron microscopy (SEM) was used to investigate the microstructures of the bioinks. After fixation with 4% *v*/*v* PFA, the samples were dehydrated using ethanol (50–95% *v*/*v*) and lyophilized. They were then coated with platinum at 15 mA for 45 s using a K575X sputter coater (Quorum Technologies, Ashford, UK) and imaged with cold field emission SEM (FE-SEM, SU8220, Hitachi, Tokyo, Japan) at 5 kV and 10 μA.

### 2.5. Two-Dimensional/Three-Dimensional Printability Tests

Two-dimensional and three-dimensional printability tests of the bioinks were performed using a custom-designed three-dimensional bioprinter. The bioprinter was composed of a 3-axis stage, a multi-head dispensing module, and an enclosure equipped with a temperature controller (Figure S1, Supporting information). The X-/Y- and Z-axis stages had resolutions of 250 nm and 500 nm, respectively, and the dispensing module was composed of mechanical dispensers (SMP-III; Musashi Engineering Inc., Tokyo, Japan) and a pressure controller (ML-808GX; Musashi Engineering Inc.). For the printability tests, the bioinks were printed at a dispensing rate of 0.5735 μL/s using a 200 μm nozzle and mechanical dispenser. Line printability tests were performed using the prepared bioinks. After printing the line patterns at printing speeds of 10–320 mm/min, the patterns were imaged using a microscope (Dino-Lite, Hsinchu, Taiwan), and their widths and heights were measured using ImageJ software (version 1.53t, NIH, Bethesda, MD, USA). The aspect ratios of the printed lines were calculated by dividing the line heights by widths. To evaluate the 2D printability of the dECM bioinks, grid patterns with pore sizes of 300–1000 μm were printed at a printing speed of 160 mm/min and layer thickness of 100 μm. After imaging with the microscope (Dino-Lite), the printed pore areas were measured using ImageJ software (NIH), and their pore fidelities were calculated using the following equation:

$$Pore\;fidelity\left(\%\right)=\frac{Printed\;pore\;area}{Designed\;pore\;area}\times 100$$

For the 3D printability test, square patterns (100 μm layer thickness and 8 × 8 mm^2^ size) were bioprinted and stacked. After printing the 1–20-layered structures, their side views were imaged, and stacked thicknesses were measured using ImageJ software (NIH).

### 2.6. Cell-Laden Bioink Preparation and 3D Bioprinting

PMHs and HUVECs were used to confirm the cytocompatibility of the developed dECM gBioink. PMHs from an 8-week-old male C57BL/6 mouse were isolated using two-step collagenase perfusion [29]. The viability of the isolated PMHs was investigated using a trypan blue exclusion test and was confirmed to be more than 90%. HUVECs (Lonza, Basel, Switzerland) were cultured in EGM™-2 BulletKit™ (Lonza) in an incubator at 37 °C under 5% CO_2_. The RFP expressing HUVECs (Angio-Proteomie, Boston, MA, USA) was used for the morphological analysis. The HUVECs were subcultured when the confluency was 80%, and the passage of less than 8 was used in the study.

To prepare cell-laden bioink, 1 × 10^7^ cells/mL of PMHs or 1 × 10^6^ cells/mL of HUVECs were gently mixed with the prepared bioink solutions (gelatin-based bioink, collagen hydrogel, traditional dECM bioink, and dECM gBioink). Subsequently, the cell-laden bioinks were loaded into 1 mL syringes and installed in a custom-designed 3D bioprinter (Figure S1, Supporting Information). Before loading into the bioprinter, the gelatin-based bioink and dECM gBioink were incubated at 4 °C for 10 min for inducing thermal crosslinking of the gelatin. For HUVEC patterned lobule structure printing, polycaprolactone (PCL) was printed as a fabricated lobule structure frame at a 50 mm/min printing speed, 120 kPa of pressure maintained with the pressure dispenser (ML-808, Musashi engineering), and 90 °C maintained with the temperature controller (TCU-02, Musashi engineering). Then, the HUVEC-laden bioink was used to print a line pattern with a 200 μm width using a 200 μm nozzle. Lastly, the PMH-laden bioink was printed to fill the PCL frame using a 200 μm nozzle. For the structures with PMHs only or HUVECs only, only the PMH-laden bioink or HUVEC-laden bioink was printed, respectively. Finally, the following were fabricated: HUVEC-patterned lobule structure using the traditional dECM bioink (EC/PH/dECM-T), HUVEC-patterned lobule structure using the dECM gBioink (EC/PH/dECM-gB), PMH-only structure using the traditional dECM bioink (PH/dECM-T), PMH-only structure using the dECM gBioink (PH/dECM-gB), HUVEC-only structure using the traditional dECM bioink (EC/dECM-T), and HUVEC-only structure using the dECM gBioink (EC/dECM-gB).

After printing, the gelatin-based bioink and dECM gBioink groups were crosslinked for 20 min using a thrombin solution (10 U/mL), and the dECM gBioink group was further crosslinked with incubation at 37 °C for 30 min. The traditional dECM bioink and collagen hydrogel groups were thermally crosslinked at 37 °C for 30 min. Bioprinted and crosslinked PMH-laden structures were cultured in William media (Thermo Fisher Scientific) supplemented with hepatocyte maintenance supplements (CM 4000; Thermo Fisher Scientific), 10% *v*/*v* fetal bovine serum (Capricorn, Ebsdorfergrund, Germany), and 6 μg/mL aprotinin (Sigma) and HUVEC-laden structures were cultured in EGM™-2 BulletKit™ (Lonza) in an incubator at 37 °C under 5% CO_2_. A 1:1 mixture of PMHs culture medium and HUVECs culture medium was used for the hepatic lobule structure culture. All animal experiments were approved by the Institutional Animal Care and Use Committee of the UNIST (IACUC protocol number: UNISTIACUC-20-50).

### 2.7. Cytocompatibility Test

Cytocompatibilities of each bioink were investigated using PMHs and HUVECs. The bioprinted PMH- or HUVEC-laden structures were cultured for two weeks or one week, respectively. To evaluate the cytocompatibility of the dECM bioinks, a live/dead cell viability test kit (Thermo Fisher Scientific) was used according to the manufacturer’s instructions. Briefly, after washing twice with PBS (Bioneer), the samples were covered with a staining solution (0.5 μg/mL of calcein AM and 2 μg/mL of ethidium homodimer-1) and incubated for 40 min at room temperature (approximately 21 °C). After imaging with a fluorescence microscope (DM2500, Leica), the numbers of live (green) and dead (red) cells were counted. The cell viability was calculated as the ratio of the number of live cells to the total number of cells. The alamarBlue™ Cell Viability Reagent (Thermo Fisher Scientific) was used to analyze the proliferation ratio of HUVECs in each bioink according to the manufacturer’s protocol. Briefly, printed structures were incubated in a 1:10 ratio of the alamarBlue cell viability reagent and culture medium for 2 h. The fluorescence was measured using a Synergy NEO2 Hybrid Multi-Mode microplate reader (Bio-Tek, Winooski, VT, USA) at 544 nm excitation and 590 nm emission wavelengths and normalized using the value of day 1. For the morphological analysis of HUVECs, fluorescence images of RFP expressing HUVECs within the bioinks were acquired using a fluorescence microscope (DM2500, Leica). The cell length and aspect ratio of HUVECs were calculated using ImageJ software (NIH).

Hepatic functionality was investigated regarding albumin secretion, urea secretion, and CYP1A2 activity. For the secreted albumin and urea assay, the culture medium was refreshed at each time point and collected after 24 h incubation. The albumin assay was conducted using a mouse albumin ELISA kit (Koma Biotech, Seoul, Republic of Korea) according to the manufacturer’s instructions. Briefly, 100 μL of culture medium was collected in a 96-well assay plate coated with goat anti-mouse albumin antibody. Horseradish peroxidase-conjugated detection antibody was added to the plate followed by a TMB solution. The optical density was measured at a 450 nm wavelength using a microplate reader (SpectraMax Plus 384 Microplate Reader, Molecular Devices, San Jose, CA, USA). A QuantiChrom urea assay kit (BioAssay Systems, Hayward, CA, USA) was used according to the manufacturer’s instructions to measure the amount of urea in the collected medium. Briefly, 5 μL of the collected culture medium was mixed with the assay reagents (200 μL). After 20 min of incubation, the optical density was measured at a 490 nm wavelength using a microplate reader. For the lobule structure analysis, the measured albumin and urea secretion levels were normalized using the initial number of printed PMHs. Finally, the CYP activities of the bioprinted samples were evaluated by adding 3-methylcolanthrene (3-MC; 1 μg/mL in the culture medium) and incubating the samples for 48 h to activate CYP1A2. The 3-MC-containing medium was changed every 24 h, and a medium containing dimethyl sulfoxide (DMSO; Sigma) was used as the control group. Subsequently, the P450-Glo CYP1A2 assay system (Promega, Fitchburg, WI, USA) was used according to the manufacturer’s instructions to analyze the CYP activity. Briefly, the bioprinted construct was incubated with luciferin-1A2 (6 μM) and salicylamide (3 mM) in PBS for 1 h at 37 °C and 5% CO_2_. The incubated solution (25 μL) was reacted with 25 μL of luciferin detection reagent in a 96-well plate for 20 min at room temperature (approximately 21 °C). The luminescence of the samples was measured using a microplate reader (Synergy NEO2 Hybrid Multi-Mode Reader; Bio-Tek).

For the drug toxicity test, the printed lobule structures were cultured for 7 days, and incubated in culture medium with 1, 2, 4, and 8 mM of acetaminophen (APAP; Sigma) for 48 h. An APAP stock solution was prepared using DMSO, and the final concentration of DMSO was less than 0.5%. After APAP treatment, the CellTiter-Glo^®^ 3D cell viability assay kit (Promega) was used to measure the viability of each hepatic lobule structure according to the manufacturer’s protocol. The luminescence was measured using a microplate reader (Synergy NEO2 Hybrid Multi-Mode Reader; Bio-Tek) and normalized with the value of the non-treated group.

### 2.8. Statistical Analysis

The data are presented as average values with standard deviations (SDs). The statistical analysis was conducted using a one-way analysis of variance (ANOVA) followed by Tukey’s multiple comparison test. Statistical significance was denoted as * *p* < 0.05, ** *p* < 0.01, and *** *p* < 0.001, and *p* > 0.05 was used to denote no significant differences (ns). The image analysis was conducted using the representative images acquired with the microscope.

## 3. Results

### 3.1. Biochemical and Histological Assays of the Decellularized Liver Tissue

**Figure 1:**
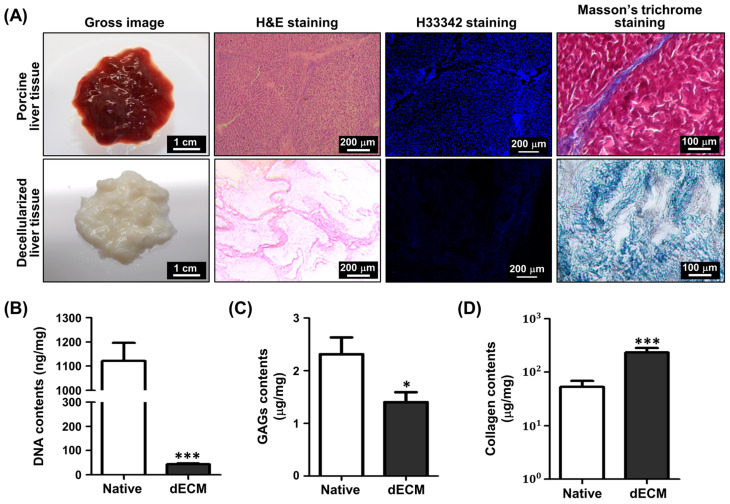
Analysis of liver decellularized extracellular matrix (dECM) using histological and biochemical assays. (**A**) Gross images and microscopic images after H&E, H33342, and Masson trichrome staining of native and decellularized liver tissue. Biochemical assays for (**B**) DNA, (**C**) glycosaminoglycans (GAGs), and (**D**) collagen in the native liver and the liver dECM (*n* = 5; * *p* < 0.05; *** *p* < 0.001 compared with the native group).

The degree of decellularization was assessed using histological and biochemical analyses (Figure 1). The gross images showed that the native porcine liver tissues turned white during decellularization (Figure 1A). In the H&E staining results, eosin-stained ECM components could be clearly observed in the decellularized liver tissue, whereas cellular components (purple) were scarce. The removed cells were confirmed with the cell nucleus staining using H33342. After decellularization, the most of cells were removed, and much less fluorescence was detected in the decellularized liver tissue. The Masson trichrome staining result showed that the blue-stained collagen successfully remained after the decellularization. For the biochemical analysis, the DNA, GAGs, and collagen contents of the dECM materials were measured (Figure 1B). Quantification of the DNA content showed that approximately 96.13% of the DNA was removed during the decellularization process, and only approximately 43.44 ± 3.11 ng/mg remained in the decellularized tissue. Moreover, 1.41 ± 0.25 and 234.55 ± 47.08 μg/mg of GAGs and collagen were preserved in the decellularized tissues, respectively.

### 3.2. Rheological and Mechanical Properties of the dECM gBioink

**Figure 2:**
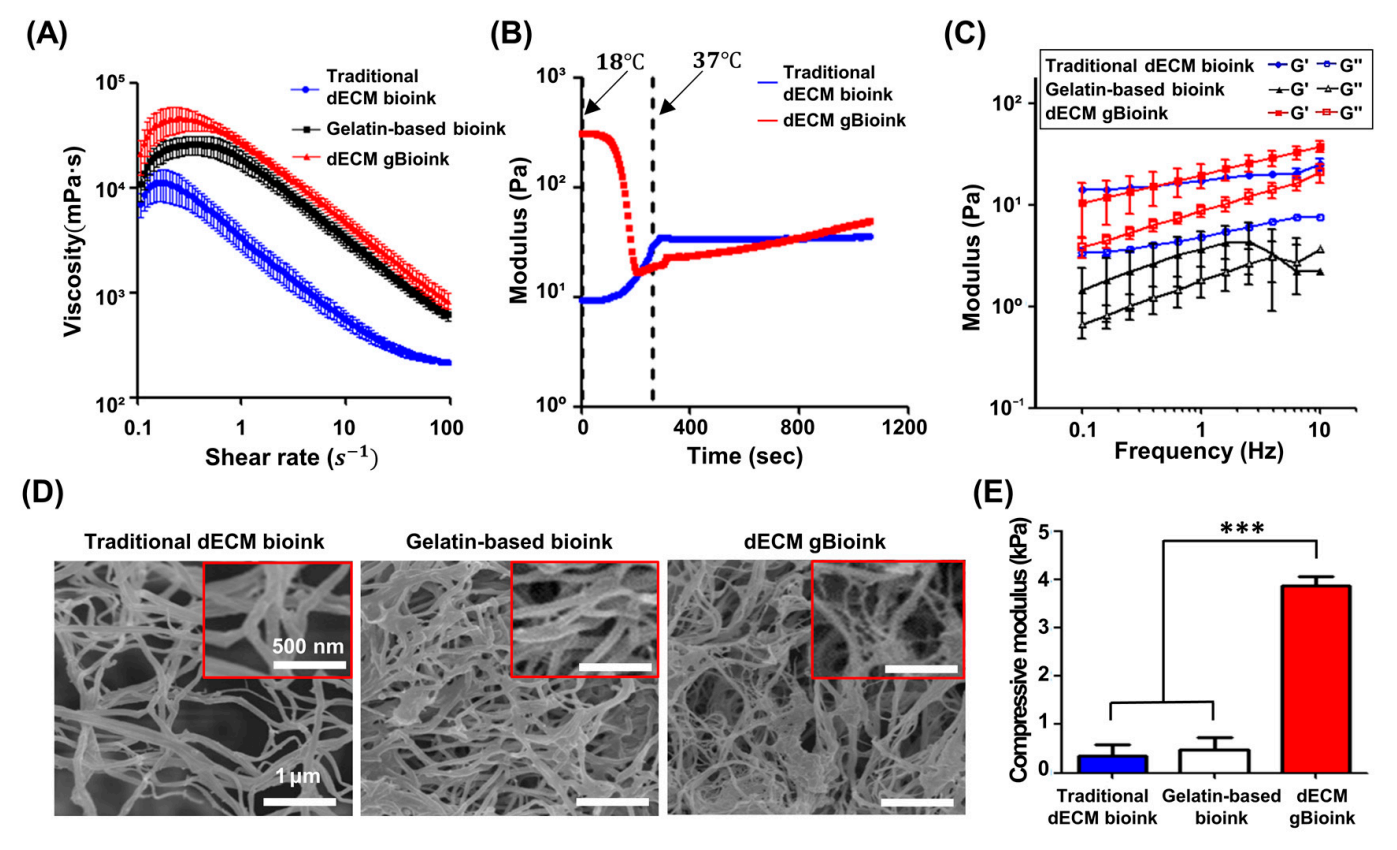
Mechanical characterization of the bioinks. (**A**) Viscosity, (**B**) thermal sweep analysis results, and (**C**) storage (G′) and loss (G″) modulus of traditional dECM bioink, gelatin-based bioink, and dECM gBioink measured with rheological testing (*n* = 3). (**D**) SEM images showing the microstructure of the bioinks. The red boxes show enlarged images. (**E**) The compressive modulus of the bioinks. (*n* = 3; *** *p* < 0.001).

The rheological properties of the prepared bioinks were investigated (Figure 2A–C). The dECM gBioink had the highest viscosity, approximately 3.20–8.38 times and 1.07–2.12 times higher than the traditional dECM and gelatin-based bioinks, respectively (Figure 2A). A shear thinning property, wherein the viscosity decreased as the shear rate increased, was observed in all bioink groups. The thermal sweep analysis results showed that the dECM gBioink could be thermally crosslinked (Figure 2B). Owing to the gelatin material in the ink, the modulus of the dECM gBioink decreased as the temperature increased, but it increased at approximately 37 °C. Similarly, the modulus of the traditional dECM bioink increased at approximately 37 °C. The frequency sweep analysis showed that the storage modulus of the crosslinked dECM gBioink and traditional dECM bioink were higher than their loss modulus in the applied frequency range (0.1–10 Hz), indicating that the crosslinked hydrogel stably maintained structure under the dynamic environment (Figure 2C). In contrast, the gelatin-based bioink had a higher loss modulus than the storage modulus at the applied frequency range of 3.98 Hz or higher.

The microstructures of the crosslinked bioinks were investigated using SEM imaging (Figure 2D). Nanofibrous structures were observed in all bioink groups, and the dECM gBioink showed interpenetrating networks. Moreover, the dECM gBioink had the highest compressive modulus, which was approximately 8.24–10.73 times higher than that of the traditional dECM and gelatin-based bioinks (Figure 2E). No significant differences were observed between the gelatin-based and traditional dECM bioink groups.

### 3.3. Two-Dimensional and Three-Dimensional Printability of the dECM gBioink

**Figure 3:**
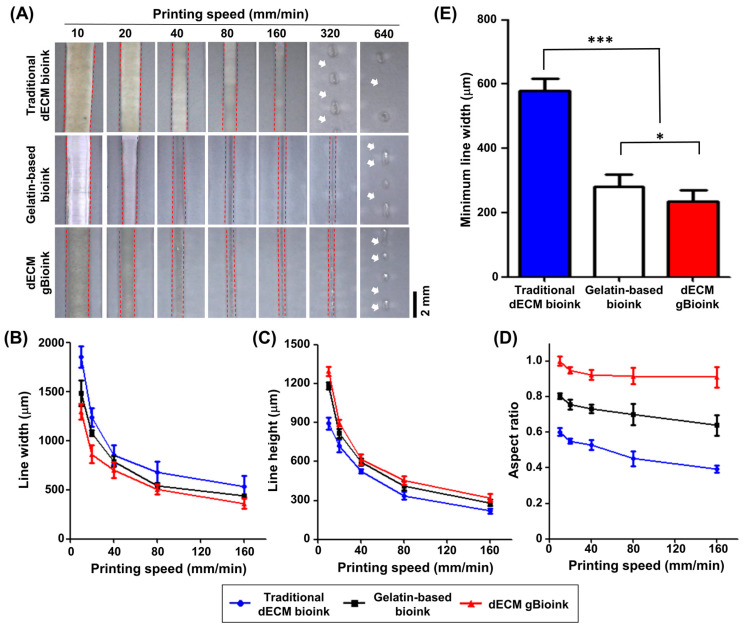
Two-dimensional line printing results of bioinks. (**A**) Microscopic images of the printed 2D line using the traditional dECM bioink, gelatin-based bioink, and dECM gBioink with various printing speeds. White arrows indicate broken areas on the printed lines. Red dashed lines indicate the edges of the printed line. The (**B**) corresponding line widths, (**C**) heights, and (**D**) aspect ratios were measured using microscopic images. (**E**) The minimum line widths of the bioinks. (*n* = 5; * *p* < 0.05; *** *p* < 0.001).

The 2D printability of the dECM bioinks was investigated using line patterning. Among the bioinks (traditional dECM bioink, gelatin-based bioink, and dECM gBioink), the width and height of the printed lines progressively decreased as the printing speed increased (Figure 3A). The dECM gBioink and gelatin-based bioink groups obtained continuous line extrusion up to a printing speed of 320 mm/min, whereas the traditional dECM bioink formed a discontinuous line. The width and height of the printed lines decreased exponentially in all bioink groups as the printing speed increased (Figure 3B,C). Among them, the line pattern in the dECM gBioink group had the smallest width and largest height. This tendency was more clearly observed in the aspect ratios calculated using the measured width and height. The highest aspect ratios were observed in the dECM gBioink group, which were approximately 1.15–2.2 times higher than those in the other groups (Figure 3D). In contrast, the traditional dECM bioink group exhibited the lowest aspect ratios. Additionally, the dECM gBioink exhibited the highest resolution among the three bioink groups (Figure 3E). The minimum printable line width of the dECM gBioink using a 200 μm nozzle was approximately 235.56 ± 34.99 μm, which was 0.83 times and 0.41 times smaller than those of gelatin-based bioink and traditional dECM bioink groups, respectively.

**Figure 4:**
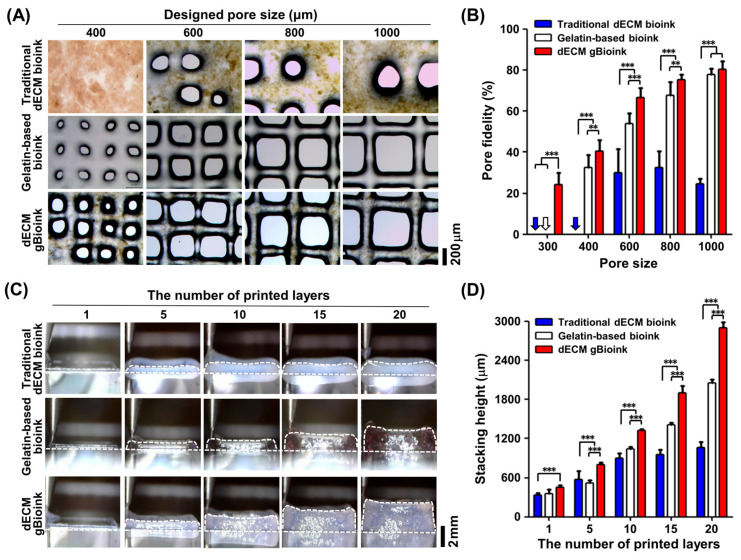
Printability test of bioinks. The 2D grid patterning test was evaluated with (**A**) microscopic images and (**B**) pore fidelities by pore sizes. The pore fidelity was calculated by dividing the measured pore area by the designed area. (**C**,**D**) The 3D layer stacking test was conducted using the bioinks. (**C**) Microscopic images showing the side view of the printed structure with the various number of stacking layers. The white dashed line indicates the outline of printed structures. (**D**) The stacking heights of the multi-layered structures were calculated using the microscopic image in (**C**). (*n* = 5; ** *p* < 0.01; *** *p* < 0.001).

The printability of each bioink was analyzed using the grid patterning and stacking test (Figure 4). Grid patterns with 400–1000 μm pores were printed to investigate the 2D patterning abilities of the dECM bioinks. As shown in the microscopic images (Figure 4A), grid patterns with the designed pore sizes could not be obtained using the traditional dECM bioink at a 400 μm size of the pore. In the other two groups, grid patterns similar to the designed rectangular pore shape were obtained as the pore size increased, and the best patterning result was achieved using the dECM gBioink group. This trend can be clearly observed in the pore fidelities of the grid patterns (Figure 4B). The dECM gBioink and gelatin-based bioink groups showed 1.8–3.27 times higher pore fidelity than the traditional dECM bioink group. Specifically, the dECM gBioink group had higher pore fidelity than the gelatin group, and statistical significance was also observed in pore sizes below 800 μm groups.

Finally, a stacking test was performed to investigate the 3D printability of the dECM bioinks. The dECM gBioink could be stably stacked in up to 20 layers in a square pattern (Figure 4C). In contrast, in the traditional dECM bioink group, the structure collapsed when 10 or more layers were stacked. Among the three groups, the dECM gBioink obtained the highest stacking height, followed by the gelatin-based bioink group (Figure 4D). Statistical significance was also observed for these differences.

### 3.4. Cytocompatibility Test of the dECM gBioink

**Figure 5:**
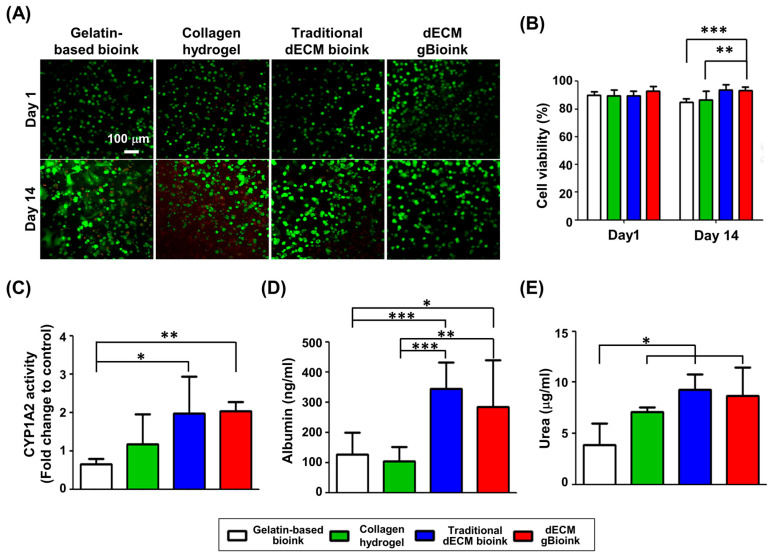
Viabilities and hepatic functionalities of primary mouse hepatocytes (PMHs) in bioprinted structures. (**A**) Live/dead staining images of bioprinted PMH-laden bioinks (green: live cells; red: dead cells). (**B**) The viabilities on day 1 and day 14 were calculated using the live/dead staining images. Measured (**C**) CYP1A2 activities and (**D**) albumin and (**E**) urea secretions of the bioprinted hepatocytes on day 7. (*n* = 5; * *p* < 0.05; ** *p* < 0.01; *** *p* < 0.001).

The cytocompatibility of bioinks was evaluated using PMHs and HUVECs. The collagen hydrogel, which is a commonly applied biomaterial for hepatic cell culture scaffolds, was used as the control group. During the 14-day culture, a slight increase in the number of dead cells was observed in the live/dead staining results of the gelatin-based bioink and collagen hydrogel groups (Figure 5A). In contrast, no significant difference was observed by time in the traditional dECM bioink and dECM gBioink groups. This trend was more clearly observable in their quantified cell viabilities (Figure 5B). The traditional dECM bioink and gBioink groups maintained high cell viability (>90.33%) for 14 days, whereas those of the gelatin-based bioink and collagen groups showed a slight decrease on day 14.

CYP activity and albumin/urea secretion were measured to evaluate the hepatic functions of the encapsulated and printed PMHs. Among the four groups, the dECM gBioink and traditional dECM bioink showed the highest CYP1A2 activity (Figure 5C). The activities of these two groups were approximately 1.75–3.53 times higher than those of the collagen hydrogel and gelatin-based bioink groups, and statistical significance was also observed. A similar tendency was observed in the albumin and urea secretion measurement results. Among the four groups, the dECM gBioink and traditional dECM bioink groups showed the highest albumin and urea secretions, without a significant difference between the two groups (Figure 5D,E). The albumin secretions in the two dECM-containing groups were approximately 2.24–3.28 times higher than those in the gelatin-based bioink and collagen groups, and statistical significance was also observed. Similarly, the urea secretions in the two dECM groups were approximately 2.24–2.42 times higher than that in the gelatin-based bioink group. Compared to the collagen hydrogel group, the urea secretions in the dECM-containing groups were slightly higher, but no statistical significance was observed. These results indicate that the dECM gBioink has good hepatocyte compatibility, which was comparable to the traditional dECM bioink.

The cytocompatibility of HUVECs in gelatin-based bioink, collagen hydrogel, traditional dECM bioink, and dECM gBioink was evaluated using cell morphology and a proliferation test (Figure S2, Supporting Information). Red fluorescence images showed the morphology of RFP expressing HUVECs (Figure S2A, Supporting Information). The dECM gBioink and gelatin-based bioink groups had slightly increased length and aspect ratio of HUVECs than the collagen hydrogel and traditional dECM bioink groups (Figure S2B,C, Supporting Information). Specifically, the dECM gBioink showed a 1.80-fold longer length and a 1.39-fold increased aspect ratio compared to the traditional dECM bioink. The normalized proliferation ratio of HUVECs in every bioink group was increased during the culture period (Figure S2D, Supporting Information). The gelatin-based bioink and dECM gBioink had a 1.12- to 1.31-fold higher proliferation ratio than other groups on day 7.

### 3.5. Liver Lobule Structure Printing Using the dECM gBioink

**Figure 6:**
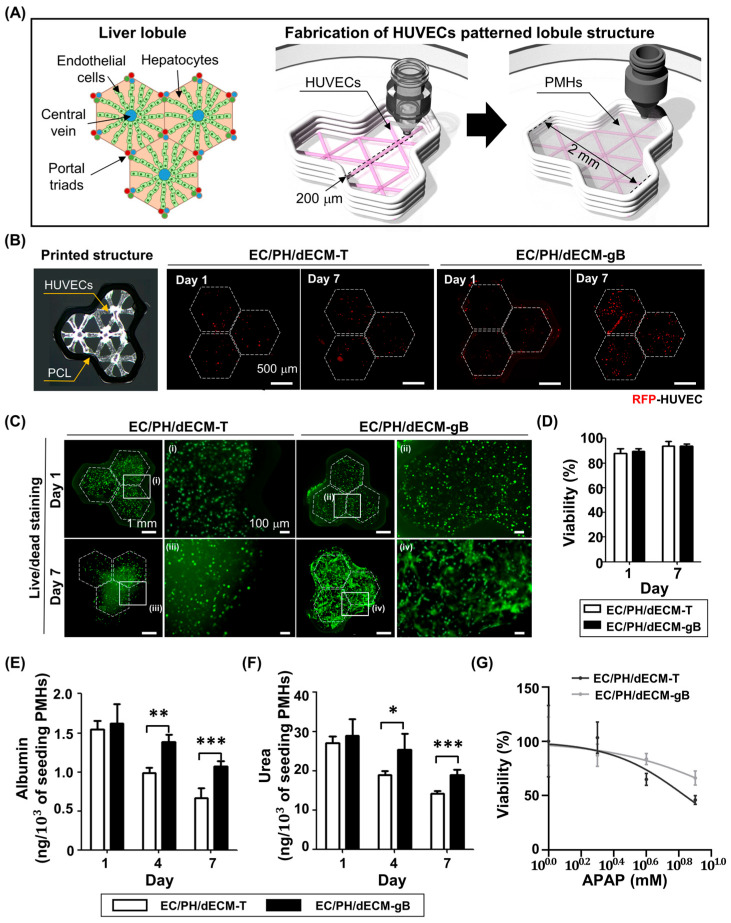
Fabrication of HUVEC-patterned liver lobule structures and hepatic functionality test. (**A**) Schematic illustration of the liver lobule and fabrication process for the HUVEC-patterned lobule mimetic structure. (**B**) Bright-field image of printed PCL frame and HUVEC line pattern. Fluorescence images showing RFP-expressing HUVECs within the patterned lobule structure using traditional dECM bioink (EC/PH/dECM-T) and dECM gBioink (EC/PH/dECM-gB). (**C**) Live/dead staining images of the EC/PH/dECM-T and EC/PH/dECM-gB groups. In the left images, dotted lines indicate the area of PCL, and the white box area was enlarged in the right image. (**D**) The viability of the printed lobule structure was calculated using the fluorescence images in (**C**). The secreted (**E**) albumin and (**F**) urea from the lobule structures were measured for 7 days. (**G**) The dose-dependent viability of the fabricated lobule structures after acetaminophen (APAP) treatment. (*n* = 5; * *p* < 0.05; ** *p* < 0.01; *** *p* < 0.001).

Liver lobule mimic structures were printed using the traditional dECM bioink and dECM gBioink (Figure 6). To fabricate the liver lobule structure, HUVEC-encapsulated bioinks were patterned, and then the PMH-laden bioinks were filled within hexagonal PCL structures (Figure 6A). The bright field image showed a printed HUVECs line, and the fluorescence images showed the morphology of RFP expressing HUVECs within the lobule structure (Figure 6B). For 7 days, the dECM gBioink group demonstrated alignment of HUVECs along the pre-patterned structure, whereas the traditional dECM bioink exhibited a dispersed pattern structure. In addition, the number of HUVECs increased more in the dECM gBioink group than in the traditional dECM bioink group on day 7. To evaluate the cytocompatibility of the printed lobule structure, live/dead staining was conducted, and its viability was measured (Figure 6C,D). Both the traditional dECM bioink and dECM gBioink groups showed proper cytocompatibility, with the viability of PMHs and HUVECs within the lobule structure remaining above 88% for 7 days. To assess the hepatic functionality of the fabricated lobule structure with the HUVEC line pattern, the secreted albumin and urea levels and drug toxicity were analyzed (Figure 6E–G). The fabricated HUVEC-patterned lobule structure using dECM gBioink (EC/PH/dECM-gB) showed 1.59-fold and 1.29-fold improved albumin and urea secretion levels, respectively, compared to the HUVEC-patterned structure using traditional dECM bioink (EC/PH/dECM-T) on day 7 (Figure 6E,G). The enhancement degree of the secreted albumin and urea levels according to the HUVEC patterning was affected by the bioink components (Figure S3A–C, Supporting Information). In the traditional dECM bioink groups, the albumin and urea secretion levels were slightly increased in the patterned structure with the HUVEC line (EC/PH/dECM-T) compared with the PMH-only structure (PH/dECM-T). On the other hand, the patterned structure with the HUVEC line using dECM gBioink (EC/PH/dECM-gB) showed a much higher increase in albumin and urea secretion levels compared to the PMH-only structure using the dECM gBioink (PH/dECM-gB). Finally, a hepatotoxicity test was conducted to confirm the sensitivity of the toxic effect of hepatotoxicants and acetaminophen (APAP) (Figure 6G and Figure S4, Supporting Information). The viability of the printed structure decreased in a dose-dependent manner in every group. The PH/dECM-T group and PH/dECM-gB group showed a similar response to APAP treatment (Figure S4A, Supporting Information). However, Figure 6G shows that the APAP sensitivity was significantly enhanced in the EC/PH/dECM-gB group compared to the EC/PH/dECM-T group. Using the 8 mM APAP treatment, the viability of the EC/PH/dECM-gB group was 45.90 ± 4.04%, which was 0.69-fold lower than that of the EC/PH/dECM-T group. The PMH-only structures (PH/dECM-T and PH/dECM-gB) showed higher drug sensitivity compared with the HUVEC-patterned structures (EC/PH/dECM-T and EC/PH/dECM-gB). The HUVEC-only structures using both the traditional dECM bioink (EC/dECM-T) and dECM gBioink (EC/dECM-gB) had APAP dose-dependent toxicity with no significant difference between the groups. The measured viability was used to calculate IC50 values (Figure S4B, Supporting Information). The IC50 values of the PH/dECM-T group and PH/dECM-gB group were 3.40 mM and 3.09 mM, respectively. The HUVEC-patterned structure had increased IC50 values for the printed structures at 13.76 mM (EC/PH/dECM-T) and 6.89 mM (EC/PH/dECM-gB). Specifically, between the HUVEC-patterned structure groups, the EC/PH/dECM-gB group exhibited a significantly decreased IC50 value for the fabricated lobule structures compared to the EC/PH/dECM-T group.

## 4. Discussion

To date, various types of liver dECM bioinks with tissue-specific biochemical compositions have been developed for use in liver tissue engineering [30,31,32,33]. However, traditional liver dECM bioinks have weak mechanical properties and low printability, making it difficult to generate precise 3D cellular structures [34]. The low printability of traditional dECM bioinks has limited precise patterning using multiple types of cells, and their weak mechanical properties have restricted layer-by-layer processing to produce 3D structures. In this study, a novel liver dECM gBioink with significantly improved mechanical properties and printability was developed. The proposed liver dECM gBioink exhibited superior mechanical properties compared with the traditional liver dECM bioink. These enhanced mechanical properties of the dECM gBioink were highly related to its composition. The interpenetrating polymer networks in composite hydrogels significantly affect the mechanical strength of bioink [35,36]. The dECM gBioink was a composite hydrogel with nanofibrils of fibrinogen and collagen of liver dECM, and these components of the dECM gBioink enhanced the modulus. In addition, the SEM images showed that the dECM gBioink was composed of interpenetrating network structures between collagen and fibrin fibrils. Therefore, the high mechanical properties of the dECM gBioink were attributed to the dense microstructure of the material.

Incorporating a gelatin mixture into the liver dECM bioink enhanced its viscosity and significantly improved its 2D/3D printability compared to traditional dECM bioink. Ouyang et al. [37] reported that the rheological behavior of a bioink is highly correlated with the integrity of printed structures. They found that a higher viscosity of the bioink leads to better structural integrity. Accordingly, among the three bioinks prepared in this study (traditional dECM bioink, gelatin-based bioink, and dECM gBioink), the dECM gBioink exhibited the highest viscosity and best printability. 

The viscosity of the dECM gBioink was significantly enhanced, being approximately 8.38 times higher than the traditional dECM bioink and 1.27 times higher than the gelatin mixture. This improvement in viscosity directly impacted its printability. The printability test confirmed that the dECM gBioink enabled 2D patterning with a significantly higher resolution and aspect ratio than other bioinks. The viscosity also had a significant impact on multilayer stacking. Owing to the low viscosity of the traditional dECM bioink, the grid patterns were not well formed, and the 3D structures collapsed during multiple layer stacking. In contrast, the pattern printed with the dECM gBioink could be stacked in 20 or more layers.

Interestingly, dECM gBioink not only had 3D printability but also demonstrated suitable cytocompatibility of PMHs and HUVECs with the traditional dECM bioink. Many researchers have reported limitations in the in vitro culture of primary hepatocytes due to a spontaneous decrease in viability and functionality after isolation [38]. Consequently, liver dECM materials have received considerable attention because they are known to adequately support the maintenance of viability and functionality of primary hepatocytes in vitro culture [12,39,40]. Lin et al. [41] demonstrated that liver-derived dECM sheets provide an excellent environment for maintaining the in vitro hepatic function of primary hepatocytes. Aleksander et al. [42] demonstrated that the viability of primary hepatocytes could be greatly enhanced using dECM materials. Similarly, in this study, the dECM components in the developed dECM gBioink significantly improved the viability and function of primary hepatocytes. Specifically, the developed dECM gBioink also showed excellent cytocompatibility with HUVECs. In particular, the fibrin gel included in the dECM gBioink is useful for the study of angiogenesis and vasculogenesis [43]. Furthermore, the various vasculogenesis-related factors included in the dECM also contribute to HUVEC cytocompatibility [44]. In this study, HUVEC cytocompatibility, such as proliferation, increased in the traditional dECM bioink and dECM gBioink compared with the collagen hydrogel composed of atelocollagen. Specifically, the dECM gBioink, which includes both fibrin gel and dECM, showed significantly increased HUVEC proliferation and a slightly more stretched morphology compared with the collagen hydrogel and traditional dECM bioink groups. Although vascularization was not observed in this study, further research is needed to optimize HUVEC culture conditions, such as scaffold stiffness, HUVEC concentration, and regulation of growth factors, in order to enhance vascularization.

The liver lobule, which is a structural unit of hepatic tissue, consists of hepatocytes and non-parenchymal cells, and their interactions are needed to maintain hepatic metabolism. Endothelial cells are one of the representative non-parenchymal cells that are utilized to improve the functionality of in vitro hepatic tissue [45,46]. In this study, we fabricated liver lobule-like structures with precise patterning of PMHs and HUVECs using the dECM gBioink.

Furthermore, the dECM gBioink improved the hepatic functionality of the patterned structures (EC/PH/dECM-gB) compared with the traditional dECM bioink (EC/PH/dECM-T). This can be interpreted as a heterotypic interaction between PMHs and HUVECs that was enhanced in the EC/PH/dECM-gB group compared to the EC/PH/dECM-T group because of enhanced cytocompatibility with HUVECs in the dECM gBioink. Since the dECM gBioink stably maintained the printed structure and improved cytocompatibility of endothelial cells, HUVECs densely proliferated following the printed pattern using the dECM gBioink (EC/PH/dECM-gB) compared with the traditional dECM bioink (EC/PH/dECM-T). Similar results are observed in another study with different materials [47]. Based on this improved hepatic functionality, the EC/PH/dECM-gB group exhibited higher sensitivity to APAP compared with the EC/PH/dECM-T group [48]. On the other hand, both the traditional dECM bioink and dECM gBioink groups showed decreased sensitivity in patterned structure (EC/PH/dECM-T and EC/PH/dECM-gB) compared with the PMH-only structure (PH/dECM-T and PH/dECM-dE). This can be interpreted as a hepatoprotective effect observed when hepatocytes are co-cultured with endothelial cells. The hepatoprotective effect of endothelial cells on hepatotoxins such as APAP when co-cultured with hepatic cells has been previously reported [49,50]. According to a study by Massa et al., the protective effect of HUVECs resulted in higher resistance of HepG2/C3A cells to APAP toxicity when co-cultured [49]. Therefore, in further research, the lobule structure of the dECM gBioink can be applied to study the response to hepatotoxins based on the interaction between hepatocytes and endothelial cells.

At last, this dECM gBioink with excellent micro-patterning capabilities also can be developed with other decellularized tissue-derived bioinks. As a result, it is expected to be used for the precise arrangement of various non-parenchymal cells and parenchymal cells, enabling the development of functional units for various tissues.

## 5. Conclusions

In this study, a novel liver dECM bioink, called dECM gBioink, was developed by incorporating a gelatin mixture into a dECM material. The gelatin mixture significantly increased the viscosity of the dECM gBioink, resulting in considerably improved 2D/3D printability and mechanical properties. Furthermore, the dECM gBioink exhibited excellent hepatocyte and endothelial cell compatibility. As a result, the fabricated hepatic lobule structure using the dECM gBioink had enhanced hepatic functionality and structural maintenance compared with the structure using the traditional dECM bioink group. These results demonstrate that the dECM gBioink has a fabrication potential for highly functional hepatic tissues. Moreover, the bioink manufacturing strategy developed in this study may provide a useful method for developing various types of tissue-specific dECM bioinks with excellent physical and biological properties in tissue engineering studies.

## Data Availability

The data are available upon request to the corresponding author.

## Supplementary Materials

The following supporting information can be downloaded at: https://www.mdpi.com/article/10.3390/jfb14080417/s1, Figure S1: Custom-designed 3D bioprinter; Figure S2: Cytocompatibility of human umbilical vein endothelial cells (HUVECs) in the bioinks; Figure S3: Functionality test for fabricated lobule structures; Figure S4: Acetaminophen (APAP) toxicity test for fabricated lobule structures.
