# Supplementary material for: Liver dECM–Gelatin Composite Bioink for Precise 3D Printing of Highly Functional Liver Tissues
Source: J Funct Biomater. 2023 Aug 9;14(8):417. doi: 10.3390/jfb14080417 (PMC10455418; doi:10.3390/jfb14080417)
Supplement: Supplementary file 1 [file jfb-14-00417-s001.zip › jfb-2531343-supplementary.pdf]

Supporting Information

Liver dECM-gelatin composite bioink for precise 3D printing of highly functional liver tissues

Min Kyeong Kim[†], Wonwoo Jeong[†], Hyun-Wook Kang[*]

Department of Biomedical Engineering, Ulsan National Institute of Science and Technology (UNIST), Ulsan 44919, Republic of Korea.

Supplementary Figures

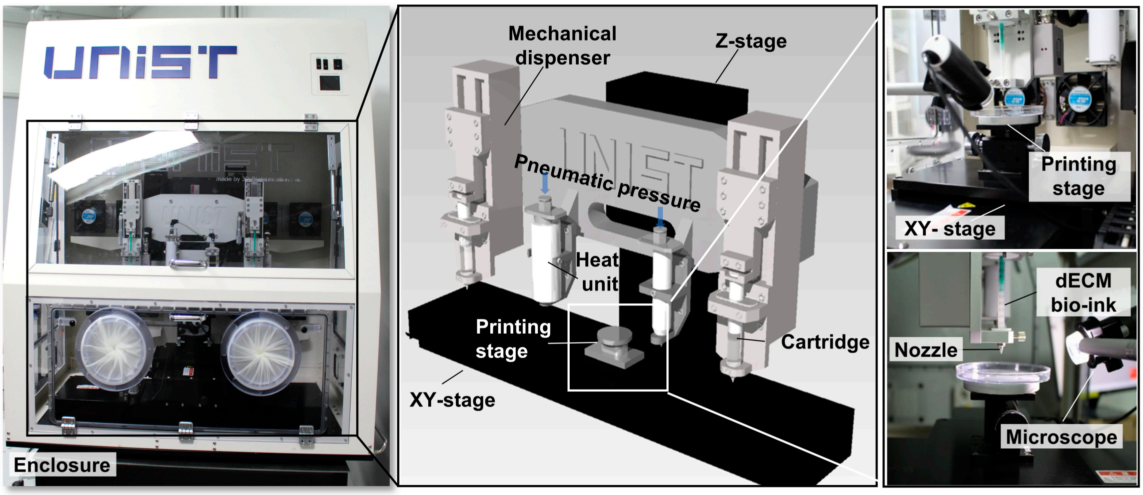

Figure S1. Custom designed 3D bioprinter. Photographs (left and right) and schematic drawing (middle) of a custom-designed 3D bioprinter composed of XYZ-stage, enclosure, microscope, heating unit, and mechanical/pneumatic dispensers.

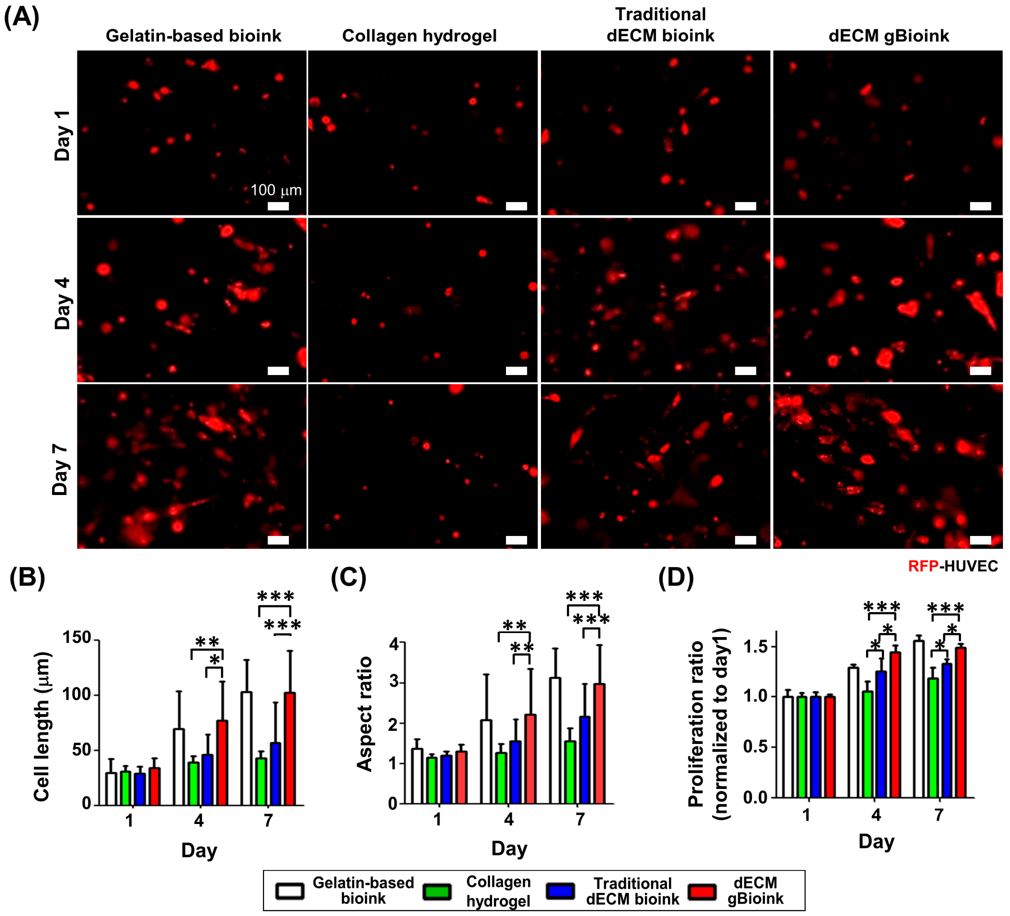

Figure S2. Cytocompatibility of human umbilical vein endothelial cells (HUVECs) in the bioinks. (A) Fluorescence image of RFP expressing HUVECs within bioinks for 7 days. To analyze morphological change of HUVECs, (B) cell length and (C) aspect ratio were measured during 7 days (n = 10). (D) Proliferation ratio was measured using alamarBlue assay (n=4). (*p < 0.05; **p < 0.01; ***p < 0.001)

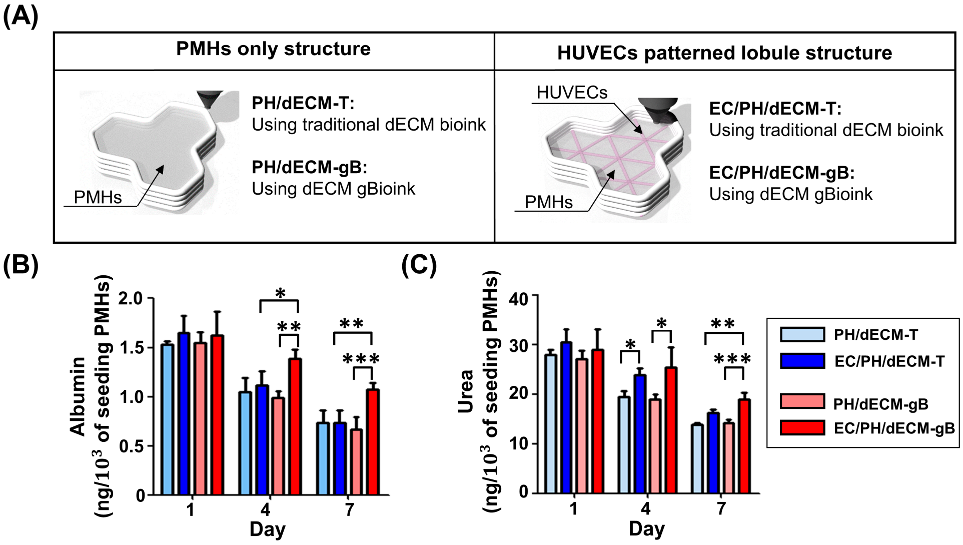

Figure S3. Functionality test for fabricated lobule structures. (A) Fabrication of liver lobule mimetic structure with PMHs only, and HUVECs patterned lobule structures. The secreted (B) albumin and (C) urea from PMHs in printed structures using traditional dECM bioink (PH/dECM-T and EC/PH/dECM-T) and dECM gBioink (PH/dECM-gB and EC/PH/dECM-gB) (n = 5).

(A)

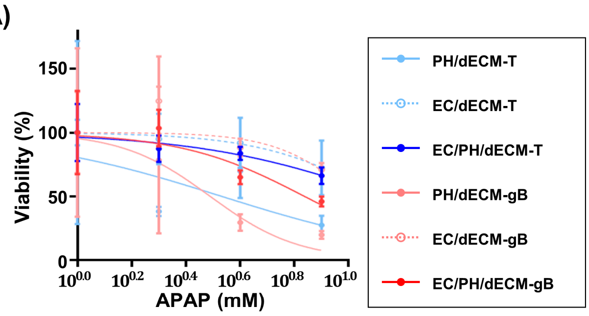

(B)

IC50 (mM)

	PH	EC	PH/EC
Traditional dECM bioink (dECM-T)	3.40	13.12	13.76
dECM gBioink (dECM-gB)	3.09	10.62	6.89

Figure S4. Acetaminophen (APAP) toxicity test for fabricated lobule structures. (A) Viability of the fabricated PMHs only, HUVECs only and patterned groups using traditional dECM bioink and dECM gBioink after APAP treatment. (n = 5). (B) The IC50 values (mM) of printed structures after APAP dose dependent treatment (n = 5).
